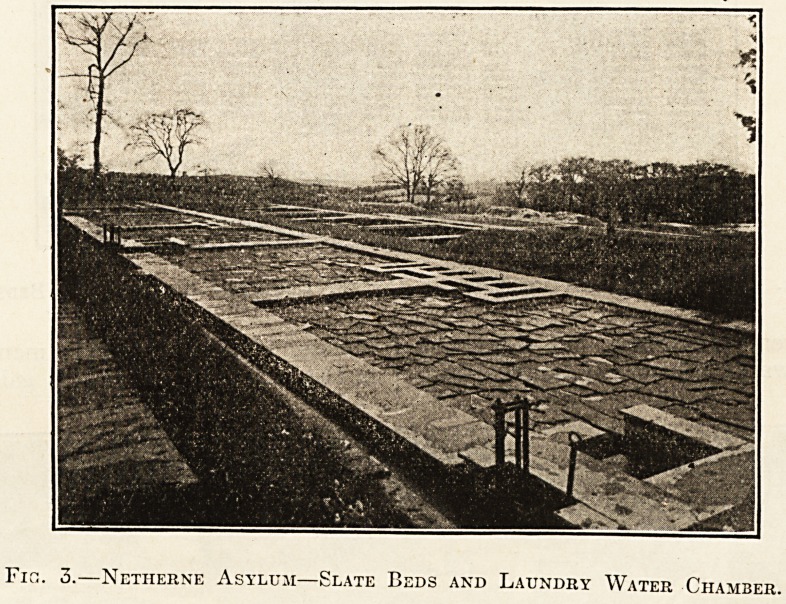# Sewage Disposal

**Published:** 1911-09-09

**Authors:** 


### SEWAGE DISPOSAL.

Modern research indicates that it is impossible to make hard-and-fast general rules for the treatment of sewage, as features present in one case are modified or totally different in another; but a primary rule may be laid down for schemes of drainage from institutions, and that is, the sewage scheme should be entire and distinct from the disposal of rain-water, which, as pointed out in a previous series, may be utilised for further service.

Investigation proves that the most satisfactory primary treatment of sewage is that in which natural methods are resorted to for the conversion of the solid matters into harmless and inodorous humus, which is a valuable manurial compost; this is the secret of the success gained by the aerobic methods of sewage disposal associated with the name of Mr. W. J. Dibdin, F.I.C., F.C.S., etc.

It has been definitely ascertained that bacteria are the natural agents chiefly employed in bringing about the indirect oxidation of sewage. The two leading

Fig. 1.—Netherne Asylum (Surrey County Council)—Building the Beds.

Fig. 2.—Netherne Asylum—Slate Construction.

questions to be considered in a scheme are the degree of purification required before the liquid can be discharged into stream, river or irrigation channels; and the economical attainment of the required degree of purification. Mr. Dibdin's system consists essentially of one or more water-tight chambers filled with superimposed layers of plates about two inches apart; these plates are thin Welsh slate slabs resting at the corners on slate cubes; no special value is ascribed to the slate itself beyond its durability and cheapness for the particular kind of work. The essential of this system is the use of horizontal plates to receive and retain the deposit of solid matters suspended in the sewage so that they are decomposed or digested by aerobic bacteria and other higher forms of life, including worms, all of which require air for their existence. Illustration No. 1 shows the method of building the beds.

The beds are filled with the raw sewage and allowed to remain for a few hours to induce quiescence, after which the liquid is slowly drawn off. It is true the solids in the sewage are not actually exposed to the air during sedimentation, but it is found that a certain amount of air is retained on the underside of the plates, and the oxygen thus available in addition to that present in the sewage is sufficient to prevent putrefaction during the comparatively short period of standing full.

As the effluent is withdrawn air circulates freely between all the layers of slate on which the solids have been deposited, thus providing the oxygen needed by the aerobic bacteria.

The result is, the ultimate residue of solids is of quite a different character from the sludge from the septic tank systems. It is of granular nature and rapidly dries on a properly constructed draining bed; when dry it resembles rich peaty mould.

At Netherne Asylum for Surrey County the average number of patients for the year is 675, and of the staff another 150 to 200; the amount of humus obtained is about six cubic yards a year, and the medical superintendent testifies that though the beds are close to his house he detects no smell. Illustrations Nos. 2 and 3 give a good idea of these beds.

These chambers or beds are generally constructed with a working depth of three to four feet where the configuration of the ground will admit of it. The residue of the solids after treatment in these beds passes out in the effluent, and it is understood that it has not been found necessary to wash out the beds or remove the deposit on the slates even after several years of operation with strong sewage.

The process before described may be called the most important in the treatment of sewage, but the effluent which is run off from the slate beds carries with it impurities which should be separated before the water is allowed to enter a stream. This object is attained by running the effluent through one or more filtering beds, which retain the matters in suspension, and the water can be run off free from impurity.

Fig. 3.—Netherne Asylum—Slate Beds and Laundry Water Chamber.

The best filtering material is acknowledged to be the extremely hard clinker from boiler furnaces more or less vitrified throughout and not only of irregular shape with rough surfaces but possessing numerous cavities on all sides. This is for the coarser filter or rather contact bed. For the fine grain beds the same material would be specified to pass a quarter-inch square mesh, and to be free from dust. After passing these contact beds the water will in all probability be considered sufficiently clear of contaminating substances to be passed on to the land or river. Sterilisation may be necessary to the final effluent by the application of chlorine in the form of a solution of chloride of lime or as a hypochlorite of sodium or of magnesium, but this is a questionable matter on account of irregularity in strength of the sterilising material and is really a question for determination on each individual instance; it is rarely required by authorities in this country.

## Figures and Tables

**Fig. 1. f1:**
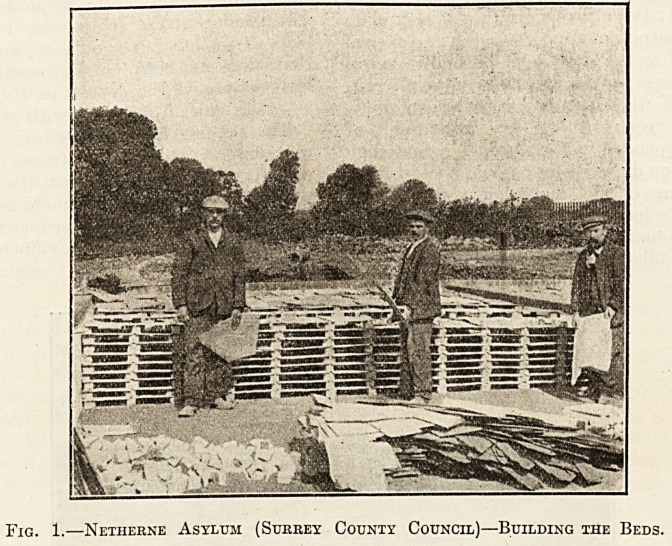


**Fig. 2. f2:**
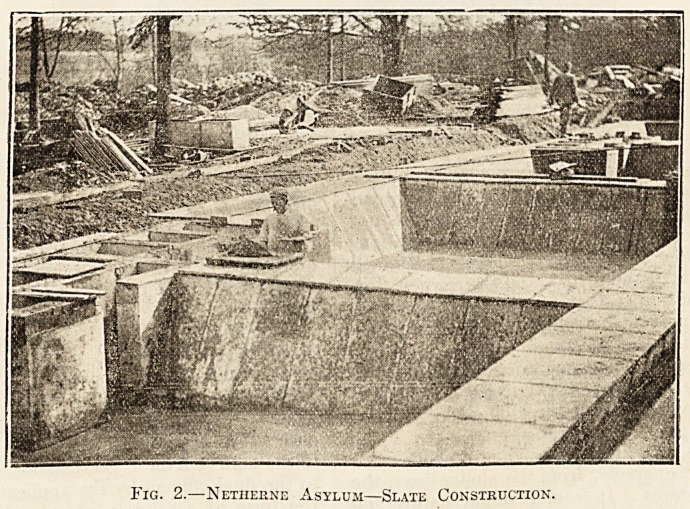


**Fig. 3. f3:**